# Activation of the Phenylpropanoid Pathway in *Nicotiana tabacum* Improves the Performance of the Whitefly *Bemisia tabaci* via Reduced Jasmonate Signaling

**DOI:** 10.1371/journal.pone.0076619

**Published:** 2013-10-25

**Authors:** Michal Alon, Osnat Malka, Galit Eakteiman, Moshe Elbaz, Michal Moyal Ben Zvi, Alexander Vainstein, Shai Morin

**Affiliations:** 1 Department of Entomology, the Hebrew University of Jerusalem, Rehovot, Israel; 2 Robert H. Smith Institute of Plant Sciences and Genetics in Agriculture, The Hebrew University of Jerusalem, Rehovot, Israel; Ghent University, Belgium

## Abstract

**Background:**

Phloem-feeding insects can manipulate plant-induced resistance and are able to suppress effective jasmonic acid/ethylene (JA/ET) defenses by the induction of inefficient salicylic acid (SA) based responses. As a result, activation of the phenylpropanoid biosynthesis pathway in transgenic plants is anticipated to cause complex interactions between phloem-feeding insects and their host plants due to predicted contradiction between two defense forces: the toxicity of various phenylpropanoids and the accumulation of SA via a branch of the activated pathway.

**Methodology/Principal Findings:**

Here, we investigated the effect of activating the phenylpropanoids pathway in *Nicotiana tabacum*, by over-expression of the PAP1 transcription factor, on the whitefly *Bemisia tabaci*, a phloem-feeding insect model. Our performance assays indicated that the over-expression made the transgenic plants a more suitable host for *B. tabaci* than wild-type (WT) plants, although these plants accumulated significantly higher levels of flavonoids. Transcription analyses of indicator genes in the SA (*PR1a*) and JA/ET (*ERF1*, *COI1* and *AOC*) pathways followed by quantification of the SA and JA hormone levels, indicated that *B. tabaci* infestation periods longer than 8 hours, caused higher levels of activity of SA signaling in transgenic plants and higher levels of JA/ET signaling in WT plants.

**Conclusions/Significance:**

Taken together, these results emphasize the important role JA/ET-induced defenses play in protecting plants from successful infestation by *B. tabaci* and likely other phloem-feeding insects. It also indicates the necessity of phloem feeders to suppress these defenses for efficient utilization of plant hosts. Our data also indicate that the defensive chemistry produced by the phenylpropanoids pathway has only a minor effect on the insect fitness.

## Introduction

Plants are in constant battle with herbivorous insects, and have evolved sophisticated defense systems to cope with insect attack, which include both induced and constitutive mechanisms [1], [2]. These defenses can influence herbivore settling, feeding, oviposition, growth and development, fecundity and fertility [3], [4].

Plant induced defenses involve activation of distinct signal-transduction pathways, in which three major plant hormones – salicylic acid (SA), jasmonic acid (JA) and ethylene (ET) are involved [5], [6]. The SA pathway is primarily activated in response to biotrophic pathogens, while the JA/ET pathways are induced in response to necrotrophic pathogens and in response to wounding and tissue-damaging by insect feeding [7], [8], although recent evidence suggests that JA can also play a role in resistance against specific types of biotrophic fungi [9]. The SA pathway regulates the expression of a wide array of defense-responses including the *Pathogenensis-related protein (PR)* coding genes [10]. In addition, the SA pathway confers a broad-spectrum resistance, the systemic acquired resistance (SAR), towards a variety of invading pathogens [11], [12]. The JA/ET pathways act to induce systemic tolerance to a range of necrotrophic pathogens and herbivorous insects [13], [14]. JA and ET can either cooperate or act as antagonists in the regulation of different stress responses (*i.e.* to pathogen attack or wounding) [15]. In the case of necrotrophic pathogens, both hormones cooperate or synergize in the activation of defense gene expression [16]. However, in the case of wound response, an antagonistic interaction between JA and ET has been described [17]. Two transcription factors, ethylene response factor 1 (ERF1) and MYC2, have been shown to participate in the regulation of these interactions. ERF1 is induced by simultaneous action of the JA and ET signaling pathways, and plays a key role in the activation of plant defenses against necrotrophic pathogen infection by regulating defense-related genes, such as *PDF1.2* [16], [18]. MYC2, on the other hand, is positively regulated by the abscisic acid (ABA) signaling pathway and functions as a transcriptional activator of genes (for example *VSP2* in *A. thaliana*) in the MYC2-branch of the JA pathway [19]. Examples of JA/ET inducible proteins that have an established or putative role in direct plant defenses include: proteinase inhibitor (PI), polyphenol oxidase (PPO), arginase, threonine deaminase, leucine amino peptidase and acid phosphatase [1], [3]. SA and JA/ET signaling pathways can also mutually affect each other largely through negative cross-talk. SA suppresses JA/ET-dependent defense gene expression, possibly through inhibition of JA synthesis or its action [20]. Similarly, JA/ET has been shown to negatively affect SA-dependent gene expression [21]. This cross-talk helps the plant to minimize fitness costs and creates a flexible signaling network that allows the plant to fine-tune its defense response to the invaders encountered [14], [22].

Plant constitutive defenses include the production of secondary metabolites that have antixenotic or antibiotic effects. One of the most widespread secondary metabolic pathways in plants involves the biosynthesis of phenylpropanoids through the shikimic acid pathway. In the starting point of the pathway, phenylalanine ammonia-lyase (PAL) catalyzes the deamination of phenylalanine to cinnamate. Coumaric acid is built by introducing a hydroxy group in the phenyl ring of the cinnamic acid, a process catalyzed by cinnamate-4-hydroxylase. Further downstream, phenylpropanoids metabolism comprises a complex series of branching biochemical pathways that provide plants with thousands of compounds, which are widely used as: structural cell components (lignin, suberin and other cell wall-associated phenolics), pigments (flavonoids, anthocyanins), immunity signals (SA) and toxins (coumarins and furanocoumarins) [23], [24]. The phenylpropanoids pathway is positively regulated by JA and its derivate methyl jasmonate (MeJA), which have been shown to induce the accumulation of PAL [18].

The possibility of manipulating the phenylpropanoid pathway, for producing plants resistant to insects, was recently explored by the development of transgenic *Arabidopsis thaliana* and *Nicotiana tabacum* that constitutively over-express the PAP1 (Production of Anthocyanin Pigment 1)/AtMYB75 or AtMYB12 regulatory proteins of flavonoids biosynthesis [25]–[27]. These transgenic plants showed significant and relative specific increase in the concentration of glycosylated anthocyanins, flavonols, and cell wall-esterified hydroxycinnamic acids when compared to wild-type plants, and also enhanced resistance to larvae of several important lepidopteran agricultural pests such as *Spodoptera frugiperda*, *Spodoptera litura* and *Helicoverpa armigera* [26]–[29].

In this study, we expanded this line of research by investigating the effect of manipulating the phenylpropanoid pathway on phloem feeding insects. In contrast to the aforementioned lepidopteran species, these insects use highly modified mouthparts (stylets) to navigate, intercellularly, through the plant cuticle, epidermis, and mesophyll and establish feeding sites in phloem sieve elements, causing comparatively little tissue damage [30]. We focused on the whitefly model *Bemisia tabaci* (Gennadius) (Hemiptera: Aleyrodidae), which is a generalist (polyphagous) species and an important global agricultural pest [31].

Our leading hypothesis was that phloem feeding insect such as *B. tabaci* will have a more complicated relationships with transgenic plants over-expressing PAP1 than lepidopteran chewing insects, due to an anticipated contradiction between two plant defense forces. On one hand, flavonoids, which are specifically over-produced in PAP1 plants [29], are translocated in the phloem [32], [33] although their concentration might be lower than in storage tissues [32], [33]. Moreover, *N. tabacum* glandular trichomes were characterized both at the transcriptomic and proteomic levels, and several flavonoids biosynthesis-related genes as well as proteins were identified [34], [35] suggesting that phloem feeding insects are likely to encounter flavonoids not only in the phloem but also in trichome exudates. Flavonoids were shown to be toxic to phloem feeders and were tightly associated with resistance of barely, wheat and cowpea to four aphid species: *Schizaphis graminum, Diuraphis noxia*, *Aphis craccivora* and *Aphis fabae* [36]–[38]. Also, O'Neill et al. [39] detected marginally significant increase in phloem concentrations of the isoflavone daidzein in response to soybean aphid (*Aphis glycines*) damage. Analyses of cassava phloem sap and honeydew excretion samples of the phloem feeding mealybug *Phenacoccus manihoti* indicated both ingestion and metabolic processing of flavonoids by the insect. In addition, significant negative correlation was observed between rutin contents of cassava infested plants and *P. manihoti* development [32].

On the other hand, PAP1 transgenic plants may accumulate high levels of SA, as one of the SA biosynthesis pathways occur via a branch of phenylpropanoids metabolism, involving side-chain shortening of cinnamic acid [23]. Accumulating evidence suggest that phloem feeding insects, such as aphids and whiteflies, can manipulate plant-induced resistance and are able to suppress effective JA/ET defenses by the induction of the inefficient SA signaling-based responses [40]–[43]. In a seminal study by Kempema et al. [44], *B. tabaci* infestation was shown to induce the SA-responsive gene transcripts, while JA and ET - responsive gene transcripts were repressed or unchanged. Furthermore, work with *A. thaliana* mutants indicated that the SA-induced defenses are beneficial to the insect and not to the plant which is better protected by JA/ET-induced defenses [42].

Testing our hypothesis was achieved by utilizing transgenic *N. tabacum* plants that constitutively over-express the PAP1/AtMYB75 transcription factor. We focused on two main objectives: First, we determined if activation of the phenylpropanoids pathway in *N. tabacum* plants alters *B. tabaci* performance. Second, we examined the effect of activating the phenylpropanoids pathway, on the basal and induced expression levels of the JA/ET and SA defense pathways, before and during 18 days of *B. tabaci* infestation.

## Results

### Biochemical and molecular characterization of PAP green and WT plants

**Figure 1:**
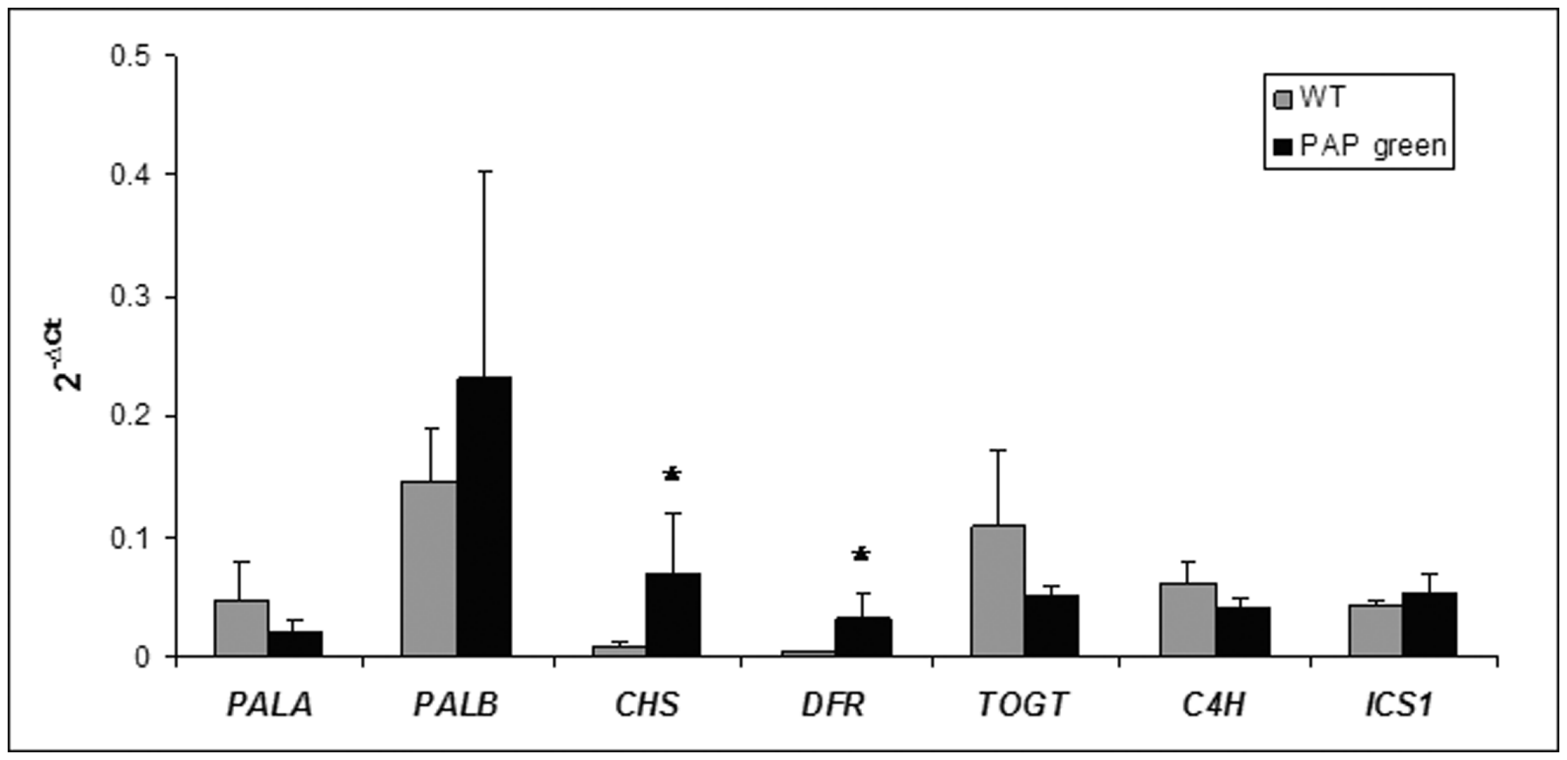
Molecular characterization of WT and PAP transgenic plants. The expression level (2^−ΔCt^ means) of genes in the phenylpropanoids pathway in WT (gray bars) and PAP green (black bars) plants. Asterisks indicate significant differences at *P*≤0.05. Error bars represent standard error of the means.

The biochemical assay indicated that the total anthocyanins content in PAP green plants was 33-fold higher than in WT plants (0.0166 vs. 0.0005 [Absorbance 530 nm/g fr wt], respectively; *F*_(1,18)_ = 121.64, *P*<0.0001). qRT-PCR analyses of *PALA* and *PALB*, revealed no significant transcriptional changes (*χ^2^*_(1)_ = 0.42, *P* = 0.51 and *χ^2^*_(1)_ = 0.047, *P* = 0.82, respectively). On the other hand, *CHS* and *DFR* (involved in flavonoids and anthocyanins biosynthesis, respectively) were significantly up-regulated in PAP green plants (*χ^2^*_(1)_ = 3.85, *P* = 0.049 for both genes). Selected genes in other branches of the phenylproapnoids pathway: *C4H*, *TOGT* and *ICS1*, did not show significant differences in their expression level between PAP green and WT plants (*χ^2^*_(1)_ = 3.85, *P* = 0.51 for *C4H*, *χ^2^*_(1)_ = 0.27, *P* = 0.27 for *TOGT*, *χ^2^*_(1)_ = 0.42, *P* = 0.51 for *ICS1*) (Figure 1).

### *B. tabaci* performance on WT and PAP green plants in no-choice experiments

**Figure 2:**
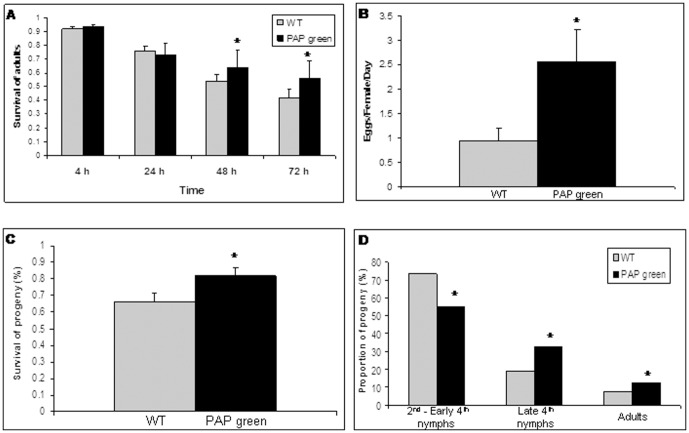
No-Choice performance experiments on WT and PAP green plants. A) Proportion of surviving adults on WT (gray bars) and PAP green (black bars) plants over a period of 72 h. B) The mean daily number of oviposited eggs per female on WT (gray bars) and PAP green (black bars) plants. C) The mean survival of progeny on WT and on PAP green plants. D) The proportion of progeny that had reached different stages of development (2^nd^ to early 4^th^ nymphs, late 4^th^ nymphs and adults) at day 18. Asterisks indicate significant differences (*P*≤0.05) between WT and PAP green plants. Error bars represent standard error of the means.

Adult's survival was significantly higher on PAP green plants than on WT plants (Figure 2A) at feeding periods longer than 24 h (*F*_(1,27)_ = 6.38, *P* = 0.0178 at 48 h and *F*_(1,27)_ = 8.84, *P* = 0.0061 at 72 h). In addition, estimations of three independent life-history traits related to reproduction, indicated increased adults and nymphs performance on PAP green plants relative to WT plants: (I) significantly higher number of eggs were oviposited (*F*_(1,33)_ = 4.76, *P* = 0.036) on PAP green plants (Figure 2B); (II) the percentage of egg hatching (egg survival) was significantly higher (*F*_(1,20)_ = 5.77, *P* = 0.026) on PAP green plants (Figure 2C); (III) the proportion of progeny that reached late 4^th^ nymphs stage and emerged as adults, 18 days after oviposition, was significantly higher on PAP green plants (χ^2^_(2)_ = 6.787, *P* = 0.033) (Figure 2D).

### Characterizing the molecular activity of the SA and JA pathways in WT and PAP green plants

**Figure 3:**
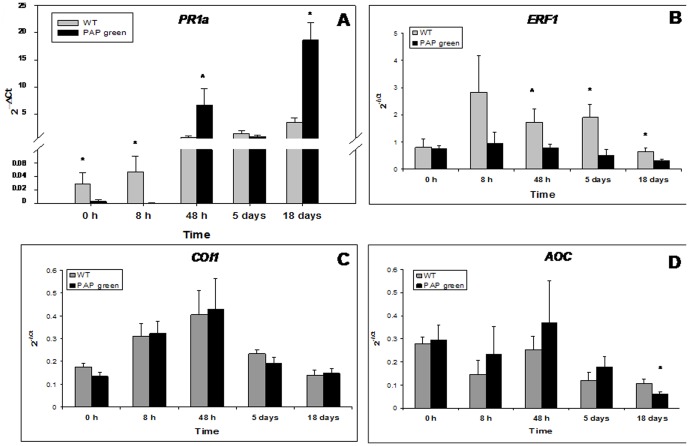
Time-course analyses of SA and JA pathway marker genes expression in WT (gray bars) and PAP green (black bars) plants before and after 8 h, 48 h, 5 days and 18 days of *B. tabaci* infestation. A) *PR1a*. B) *ERF1*. C) *COI1*. D) *AOC*. Values represent mean 2^(−ΔCt)^ ± standard error. Asterisks indicate significant differences (*P*≤0.05) between WT and PAP green plants at each time point as detected by *a priori* and orthogonal comparisons. The hat symbol indicates nearly significant differences (*P* = 0.07).

In non-infested (0 h) plants and during the first 8 h of infestation, the expression level of *PR1a* was significantly higher in WT plants than in PAP green plants (*t*_(9)_ = 2.36, *P* = 0.042 at 0 h and *t*_(10)_ = 5.53, *P* = 0.0003 at 8 h). However, during longer periods of infestation, *PR1a* transcript levels were higher in PAP green plants than in WT plants: marginally significant after 48 h of infestation (*t*_(14)_ = −1.93, *P* = 0.07) and highly significant after 18 days (*t*_(14)_ = −4.85, *P* = 0.0003). In contrast, after 5 days of infestation, no difference in *PR1a* expression was observed between WT and PAP green plants. This finding can be related to the intensive mortality of *B. tabaci* adults observed at this time point, after which new adults were added to the plants (Figure 3A).

The expression level of *ERF1* before infestation (0 h) and during the first 8 h of infestation did not differ between WT and PAP green plants (*t*_(9)_ = 0.19, *P* = 0.85 at 0 h and *t*_(10)_ = 1.36, *P* = 0.20 at 8 h). However, during longer periods of infestation, *ERF1* transcript levels were higher in WT plants than in PAP green plants: marginally significant after 48 h of infestation (*t*_(13)_ = 1.97, *P* = 0.07) and significant after 5 and 18 days (*t*_(11)_ = 2.51, *P* = 0.029 at 5 days and *t*_(13)_ = 2.09, *P* = 0.05 at 18 days) (Figure 3B). *COI1* levels did not differ between the two plant types before and after infestation (*P*>0.09). However, in both plant types, a trend of increase in the expression of *COI1* was observed until 48 h after infestation, followed by a gradual decrease until 18 days (Figure 3C). The expression levels of *AOC* were not significantly different between the two plant types (*P*>0.13), except for higher levels of expression in WT plants, 18 days after infestation (*t*_(14)_ = 2.18, *P* = 0.046) (Figure 3D).

### Chemical characterization of the SA and JA levels in WT and PAP green plants

**Figure 4:**
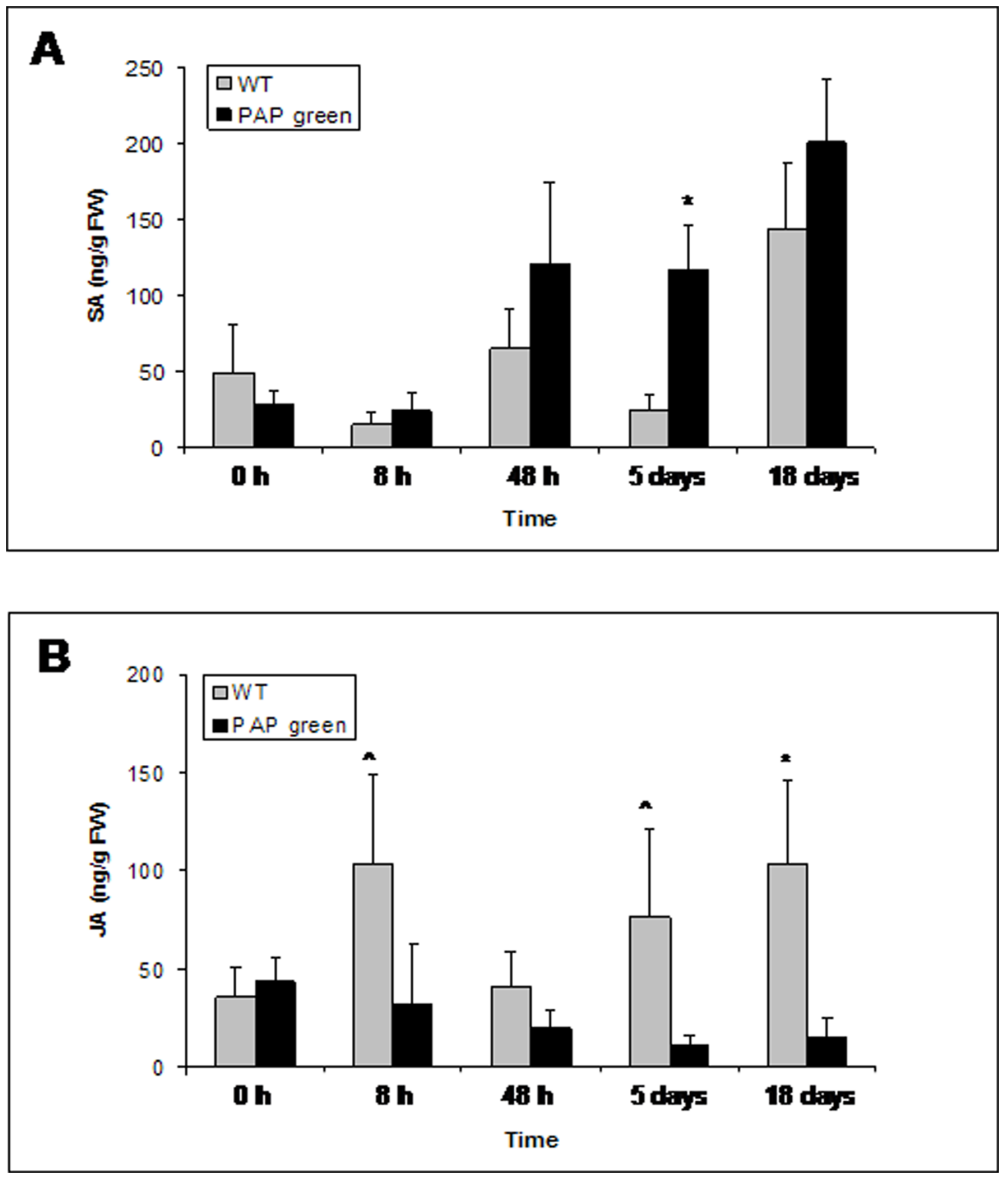
Chemical analyses of SA and JA hormone levels. A) SA levels in WT (gray bars) and PAP green (black bars) plants before and after 8 h, 48 h, 5 days and 18 days of *B. tabaci* infestation. B) JA levels in WT (gray bars) and PAP green (black bars) plants during the same infestation periods. Values are means of ng SA or JA per g fresh weight (FW) ± standard error. Asterisks indicate significant differences (*P*≤0.05) between WT and PAP green plants in each time point. The hat symbol indicates nearly significant differences (*P* = 0.07).

Independent estimations of the JA and SA pathways activity in WT and PAP green plants, before and after infestation, were obtained by gas chromatography-mass spectroscopy (GC-MS) quantification of the hormones levels. SA levels did not differ between the two plant types before infestation (*t*_(7)_ = −0.31, *P* = 0.75) and during the first 8 h of the infestation period (*t*_(7)_ = 0.075, *P* = 0.97). In contrast, after 5 days of infestation, significantly higher levels of SA were detected in PAP green plants than in WT plants (*t*_(9)_ = 1.99, *P* = 0.05) (Figure 4A). A prominent, albeit non-significant, similar trend was observed after 48 h (*t*_(8)_ = 1.26, *P* = 0.21) and 18 days of infestation (*t*_(10)_ = 1.43, *P* = 0.16). In addition, the overall plant-type effect in the three way ANOVA model (plant-type, time and biological replicate as main effects) was nearly significant (*F*_(1)_ = 3.56, *P* = 0.067).

JA levels did not differ in non-infested plants (*t*_(8)_ = 0.19, *P* = 0.84), but during the different time points after infestation, the levels were higher in WT plants compared to PAP green plants. These differences were nearly significant after 8 h and 5 days (*t*_(8)_ = −1.84, *P* = 0.07 for both time points) and highly significant after 18 days of infestation (*t*_(9)_ = −2.55, *P* = 0.014). A non significant similar trend was observed after 48 h of infestation (*t*_(8)_ = −0.55, *P* = 0.58) (Figure 4B). In addition, the overall plant affect in the three way ANOVA model was highly significant (*F*_(1)_ = 8.46, *P* = 0.006). Although induced nicotine synthesis is known to be regulated by the JA pathway [45], no significant differences were found in nicotine levels between non-infested WT and PAP green plants (*t*_(4)_ = −1.64, *P* = 0.17) and also after five days of *B. tabaci* infestation (*t*_(4)_ = −1.3, *P* = 0.26) (Figure S1 and Protocol S4 in File S1).

### *B. tabaci* development on WT and PAP green plants sprayed with SA or MeJA

**Figure 5:**
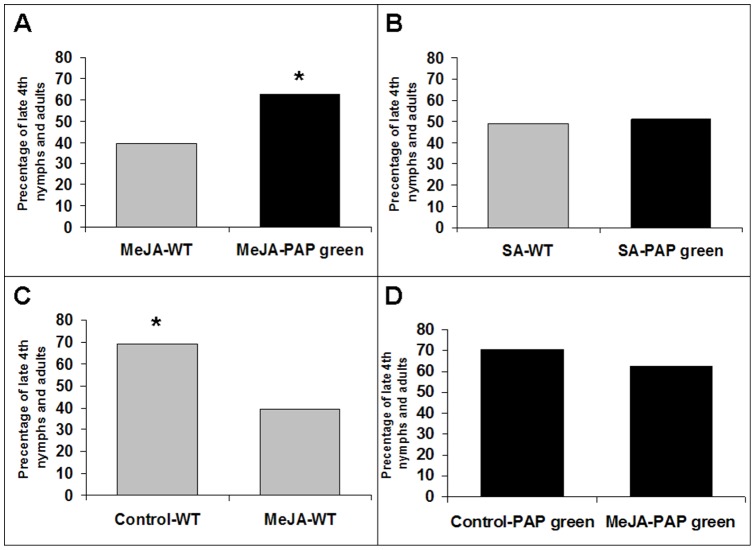
The proportion of progeny that had reached advanced stages of development (late 4^th^ nymphs and adults) by day 25 on WT (gray bars) and PAP green (black bars) plants under the three spraying regimes: SA, MeJA and control (DDW/0.015% Tween-20). Sequential Bonferroni a priori (planned) comparisons (log-likelihood ratio test) were performed on four selected pairs, using the conservative Dunn-Sidak method. A) MeJA-WT vs. MeJA-PAP green. B) SA-WT vs. SA-PAP green. C) Control-WT vs. MeJA-WT. D) Control-PAP green vs. MeJA-PAP green. Asterisks indicate significant differences (*P*≤0.05) between pairs.

As cross-talk between the JA- and SA-defense pathways is commonly associated with plant response to biotic stress, we further dissected the relative importance of the JA- and SA-defense pathways by spraying infested WT and PAP green plants with SA or MeJA (seven sprays during a three weeks period - see ‘*Materials and Methods*’) and recorded nymph developmental progression at day 25. After 25 d of infestation, 63% of the *B. tabaci* nymphs feeding on MeJA-treated PAP green plants reached the late 4^th^ nymph stage or emerged as adults while only 39% of the nymphs feeding on MeJA-treated WT plants have reached these advanced developmental stages (χ^2^_(1)_ = 7.882, *P* = 0.0050) (Figure 5A). In contrast, the percentage of late 4^th^ nymphs and emerged adults did not differ significantly between SA-treated PAP green and WT plants (51% versus 49%, respectively; χ^2^_(1)_ = 0.055, *P* = 0.81) (Figure 5B). As nymph developmental progression differed significantly between control and MeJA-treated WT plants (χ^2^_(1)_ = 14.863, *P*<0.0001) (Figure 5C) but not between control and MeJA-treated PAP green plants (χ^2^_(1)_ = 1.394, *P* = 0.24) (Figure 5D), these findings bring independent evidence both for the importance of JA-regulated defenses in resistance of WT tobacco plants to *B. tabaci* and for the low activity of JA-regulated defenses in PAP green plants (see below). Nymph developmental progression was also found to differ significantly between control and SA-treated WT plants (69% versus 49%, respectively; χ^2^_(1)_ = 9.536, *P* = 0.0020) and PAP green plants (70% versus 51%, respectively; χ^2^_(1)_ = 6.198, *P* = 0.0128). However, these findings should be interpreted cautiously as the high dose of SA (1 mM) used in our repeated spraying regime, greatly reduced the growth rate of WT and PAP green plants and was also associated with chlorosis and yellowing. As previously suggested, this likely indicates physiological or morphological changes caused by the chemical elicitor that seem not to be related to resistance but that nevertheless conduce strong effect on the herbivore fitness parameters [46], [47].

## Discussion

In this study, we investigated the effect of activating the phenylpropanoids/flavonoids pathway in a Solanaceae model, *N. tabacum*, on a phloem feeding model, the whitefly *B. tabaci*. Our no-choice performance assays indicated that activation of the phenylpropanoids pathway made the transgenic plants a more suitable host for *B. tabaci* than WT plants, although these plants accumulated significantly higher levels of flavonoids. The phenomenon was exemplified by comparing several variables, related both to the insect's survival and reproductive performance.

Enhanced herbivore performance on transgenic plants over-expressing PAP transcription factors was previously reported in at least two additional systems. Johnson and Dowd [28] over-expressed PAP1 in *A. thaliana* plants and obtained two plant phenotypes: solid purple leaves and purple-veined leaves with a green background (similar to the phenotype of our PAP green plants). While *S. frugiperda* larvae that fed on solid-purple leaves showed significant reduced feeding than those fed on WT leaves, larvae that fed on purple-veined leaves weighted significantly more than those fed on WT leaves. It was postulated that phenylpropanoids may be capable of enhancing the growth of insect herbivores up to a certain concentration (like in purple-veined leaves) but are toxic at higher concentrations (like in solid-purple leaves). Wuyts et al. [48] over-expressed PAP2, a Myb transcription factor with high similarity to PAP1 [25], in tobacco plants. PAP2 over-expression significantly stimulated the reproductive performance of the nematode *Meloidogyne incognita*. In both studies, however, the mechanism leading to the enhanced performance of the studied organism, were not explored.

To gain first insight into the fundamental differences between WT and PAP1 plants, we tested whether the two plant types differ in their induced defenses, focusing mainly on the SA and JA/ET pathways. In the SA pathway, *PR1a* transcript levels were significantly higher in WT plants before and during the first 8 h of *B. tabaci* infestation. However, at longer infestation periods, *PR1a* transcript levels were higher in PAP green plants (Figure 3A). In the JA/ET pathways, a reciprocal picture was obtained. The expression level of *ERF1* before infestation (0 h) and during the first 8 h of infestation did not differ between WT and PAP green. However, during longer periods of infestation, *ERF1* transcript levels were higher in WT plants than in PAP green plants (Figure 3B). The transcript levels of the other genes in the JA pathway, *COI1* and *AOC*, showed no significant differences between the two plant types before infestation and during the different time points after infestation, except for the higher levels of expression of *AOC* in WT plants, 18 days after infestation (Figure 3C and Figure 3D, respectively).

Independent estimations of the JA/ET and SA pathways activity in WT and PAP green plants, before and after infestation, were obtained by GC quantification of the hormones levels. SA levels were relatively low and did not differ between WT and PAP green plants before and during the first 8 h of infestation (Figure 4A), even though the transcript levels of *PR1a* were significantly higher in WT plants during this period. The reason for this is likely to be the overall low level of activity of the pathway during the first 8 h of infestation in both plant types. During longer periods of infestation, higher levels of the SA hormone were detected in PAP green plants than in WT plants (Figure 4A), which was in agreement with both the higher expression levels of *PR1a* and the better performance of *B. tabaci* on these plants. JA levels did not differ between the WT and PAP green plants before infestation, while significant higher levels of the hormone were observed in WT plants during the infestation period (Figure 4B). This was, again, in agreement with both the higher expression level of *ERF1* in WT plants, during long periods of infestation, and the reduced performance of *B. tabaci* on WT plants. The reasons why WT plants expressed higher levels of the JA hormone while PAP green plants expressed higher levels of the SA hormone after *B. tabaci* infestation, or why PAP green plants can “neutralize” the negative effect of the MeJA spraying on *B. tabaci* performance (Figures 5A and 5D) are not clear at the moment. It may relate to the tendency of the transgenic plants to accumulate higher levels of SA after insect infestation and to the well documented negative cross talk between the SA and JA/ET pathways, as previously postulated by Felton et al. [49]. It may also reflect increased metabolic flux, rather than transcriptional activation, towards SA production, without negative feedback within the phenylpropanoid pathway [50]. Alternatively, it may relate to specific interactions between uncharacterized elicitors in the *B. tabaci* saliva and components of the up-regulated phenylpropanoid pathway.

In a broader perspective, however, these results emphasize the important role JA/ET-induced defenses can play in protecting plants from successful infestation by *B. tabaci* and other phloem-feeders. Interestingly, Zarate et al. [42] also came to a similar conclusion while working on *A. thaliana* defense mutants infested with *B. tabaci*. Nymphs feeding on *cev1* plants (mutants which constitutively activate the JA defenses) showed delayed development relative to WT plants, while SA-regulated genes transcripts levels accumulated similarly in the *cev1* and WT plants. Furthermore, when *npr1* plants (mutants which lack the ability to activate the SA defenses), were treated with MeJA, the treatment accentuated the *npr1* delay in *B. tabaci* nymph development relative to the untreated *npr1* plants. Collectively, these data bring strong evidence not only for the strong negative effects of JA/ET defenses on *B. tabaci* (Figure 5C), but also for the necessity of the insect to manipulate and suppress these defenses for efficient utilization of the plant host. On the other hand, our data indicate that the defensive chemistry produced by the phenylpropanoids/flavonoids pathway seem to have only a minor effect on the insect fitness.

How do JA/ET-regulated defenses affect *B. tabaci* and other phloem-feeding insects? Originally, it was hypothesized that plant induced PIs should have only a minor effect on sap-feeding hemipterans because these insects do not carry out proteolysis of ingested proteins. However, both biochemical and molecular analyses proved this not to be true. Early studies indicated that 35S labeled cotton leaf proteins fed to *B. tabaci* were digested to free amino acids and were excreted via honeydew or used in de novo protein synthesis [51]. Cysteine-PI from rice, OC-I, was shown to affect growth and alter demographic parameters of several aphid species [52]. More recently, feeding on a wheat subtilisin-chymotrypsin inhibitor (WSCI) and a wheat cysteine-PI (WCPI), was shown to negatively effect both the survival and growth of the aphid species, *Sitobion avenae* [53]. It is important to note, however, that the observed antimetabolic activity of the dietary PIs on the aforementioned phloem-feeders, may result from effects on proteinases involved in degradation of endogenous proteins and not from inhibition of digestive proteolysis [53]. Other putative mechanisms by which induced JA/ET defenses may interfere with phloem feeders performance include the reduction of shoot quality by decreased levels of total sugar and amino acids [54] and the increased activity of enzymes such as arginase and threonine deaminase that catabolize essential amino acids [55].

In conclusion, this study is the first to provide detailed characterization of the behavioral response of polyphagous phloem-feeding-insects to activation of the phenylpropanoids pathway, and in addition, a comprehensive characterization of the transgenic plants induced defense responses to insect infestation. It is important to note that previous experiments have established that over-expression of PAP1 in transgenic plants can give protection against several important lepidopteran agricultural pests, making the technology a future “green” alternative strategy to chemical insecticides. Our findings, however, suggest that the technology might not be applicable for controlling phloem-feeding pests, which are expected to benefit from its implementation into agricultural systems.

## Materials and Methods

### Origin and maintenance of *B. tabaci* strain

The *B. tabaci* Middle East-Asia Minor 1 (MEAM1) strain (B-ref) was collected in Israel during 1987. Since its collection, the strain was reared on cotton plants (*Gossypium hirsutum* L cv Acala) under standard greenhouse conditions of 26±2°C, photoperiod of L∶D 14 h∶10 h.

### Plant material

Tobacco plants (*N. tabacum* variety Xanthi) were grown under standard greenhouse conditions (L∶D 14 h∶10 h, 26±2°C) in 6 cm diameter round pots. Transgenic tobacco plants were produced by over-expressing *PAP1*, a *MYB* transcription factor from *A. thaliana* [25] under the control of the CaMV 35S promoter. Detailed description of transgenic plant production is provided in Ben Zvi et al. [50]. The transgenic plants had primarily purple-veined leaves with a green background and were named ‘PAP green’.

Anthocyanins content in wild-type (WT) and PAP green plants were determined by the method previously reported in Alon et al. [56] (Protocol S1 in File S1). The relative activities of genes coding for key enzymes in different branches of the phenylproapnoids biosynthesis pathway: *PALA* and *PALB (Phenylalanine ammonia lyase A and B*), *C4H (Cinnamate hydroxylase)*, *CHS (Chalcone synthase)*, *DFR (Dihydroflavonol 4-reductase)* and *TOGT (UDP Glucose scopoletin glucosyltransferase)* as well as *ICS1* (*Isochorismate synthase1*), which is involved in SA biosynthesis from the shikimate pathway intermediate chorismate, were determined by quantitative real-time PCR (qRT-PCR) analyses. qRT-PCR protocol and the list of accession numbers, qRT-PCR primers and product sizes are provided in Protocol S2 and Table S1 in File S1. For further chemical analyses we relied on Malone et al. [26], which showed that transgenic tobacco plants expressing PAP1/AtMYB75 with the CaMV 35S promoter, accumulate, in addition to anthocyanins, increased levels of chlorogenic acid, caffeic acid and its other derivatives, and rutin. Moreover, comprehensive analysis of the metabolome and transcriptome of *A. thaliana* plants over-expressing the *PAP1* gene indicated that PAP1 specifically regulates flavonoid biosynthetic genes causing accumulation of cyanidin- and quercetin-type flavonoids in a relatively specific manner [29].

### *B. tabaci* egg to adult development and survival on WT and PAP green plants

All *B. tabaci* experiments were conducted in a temperature-controlled room in long day conditions (L∶D 14 h∶10 h, 26±2°C). Twenty adult couples, 48 h after emergence, were collected and spread on single WT or PAP green plants in clip cages, situated in 45×60×40 cm Perspex cage. After two days of oviposition, all adults were removed and eggs were allowed to develop. The total number of progeny, their developmental stages (second through early fourth nymphs, late fourth nymphs, empty exuvia indicating emergence), and viability status (live/dead), were recorded after 18 days. Each treatment was replicated 11–20 times. The proportions of live progeny and their developmental progression on WT and PAP green plants were compared using the log-likelihood ratio test (*G*-test). All statistical analyses conducted in this study used JMP statistical software version 7.0.1 (SAS Institute, USA) and significance level of *P*≤0.05.

### *B. tabaci* oviposition on WT and PAP green plants

For each replicate, one couple of adults, 48 h after emergence, was collected and placed in a 12×8×6 cm plastic cage. One leaf from each plant, WT or PAP green, was gently placed in the cage. Females were allowed to oviposit for 72 h. At the end of each trial, all eggs on both sides of the leaves were counted using a stereoscope, only from replicates in which the females survived the 72 h oviposition period. 7–12 replicates were conducted. The daily oviposition rate on WT and PAP green plants were compared using the Student's *t*-test.

### Survival of *B. tabaci* adults on WT and PAP green plants

Survival of *B. tabaci* adults' was compared between WT and PAP green plants in a non-choice setting, by counting the number of live and dead individuals at various time points (4 h, 24 h, 48 h and 72 h) after insect release. Twelve replicates were conducted in each time point. To test for significant differences, we used a three-way repeated-measures ANOVA in which time-point (fixed) was the repeated (within subjects) factor while plant genotype (fixed) and experiment (random) were the main (between subjects) factors. Specific means in the (plant genotype X time-point) interaction were selected a-priori and orthogonal comparisons were conducted.

### Expression of JA/ET- and SA-regulated defense genes

The level of activity of the SA and JA/ET pathways were determined in WT and PAP green plants prior to and after *B. tabaci* infestation by quantifying the expression level of the following SA/JA/ET gene markers by qRT-PCR: *PR1a* (Pathogenesis related protein 1a) which serves as a common indicator for recording the SA signaling pathway activity [57]; *ERF1* (Ethylene response factor 1) induced by simultaneous action of the JA and ET signaling pathways [16]; *AOC* (Allene oxide cyclase) involved in the JA biosynthetic pathway [58] and *COI1* (Coronatine insensitive 1) which codes for an F-box protein essential for the induced responses to jasmonates [18]. The list of gene names, accession numbers, qRT-PCR primers and product sizes is provided in Table S2 in File S1.

For determining the gene's expression levels before and after *B. tabaci* infestation, one leaf of WT or PAP green plant was placed gently into a 12×8×6 cm plastic cage. About 100 *B. tabaci* adults were aspirated into a glass tube (5 cm high×2 cm diameter) and transferred immediately to each leaf cage for 8 h, 48 h, 5 days and 18 days infestation period. Control WT and PAP green plants were not infested with *B. tabaci*. A second batch of individuals was released after five days in the 18 days infestation cages, to maintain effective infestation levels. At the end of each infestation period, adults were removed from leaves and total RNA extraction, purification, cDNA synthesis and qRT-PCR analyses were conduced as described in Protocol S2 in File S1. Significant differences in gene expression levels were tested using a one-way ANOVA model.

### Chemical characterization of SA and JA levels in WT and PAP green plants

SA and JA levels were quantified in WT and PAP green plants prior to and after *B. tabaci* infestation by gas chromatography-mass spectroscopy (GC-MS) (Protocol S3 in File S1). The experimental setting was identical to the one described above for determining the expression of JA- and SA-regulated defense genes. 5–6 biological replicates were performed for each time point. Significant differences in SA and JA amounts (ng/g leaf fresh weight) were tested by a three-way ANOVA model. Plant and time were set as fixed effects while biological replicate was set as a random effect.

### *B. tabaci* development on WT and PAP green plants sprayed with SA or MeJA

WT and PAP green tobacco plants were grown under standard greenhouse conditions (L∶D 14 h∶10 h, 26±2°C) in 6 cm diameter round pots until the third true leaf stage. 1 mM SA or MeJA (Sigma) in 0.015% Tween-20 were applied with a household sprayer. Control plants were sprayed with DDW/0.015% Tween-20 only. Spraying was conducted seven times: 24 h before *B. tabaci* infestation and twice a week for three weeks after infestation. Before spraying, the infested leaf was covered in order to exclude the possibility of a direct effect of the hormones on insect performance. Fifty adult couples (male and female), 72 h after emergence, were collected into a clip cage, 3 cm in diameter, which was attached to a third true leaf. After a 24 h oviposition period, all adults were collected and eggs were allowed to develop. The developmental rate was estimated by calculating the percentage of late fourth nymphs and empty exuvia (indicating emergence) on each plant, 25 days after infestation. Developmental progression on WT and PAP green plants under the three spraying regimes (total of six treatments, 3–6 replicates in each one) were compared using the log-likelihood ratio test (*G*-test). Sequential Bonferroni a priori (planned) comparisons were performed on four selected pairs, using the conservative Dunn-Sidak method at a specified experimental error rate of *α* = 0.05 (*α′* = 1−[1−0.05]^1/*k* = 4^). This was done in order to avoid minute *α′* values obtained when carrying out unplanned comparisons among all possible pairs of means (*k* = 6×5/2 = 15 comparisons). The four pairs were selected in order to test for two main differences in developmental performance: (I) between nymph feeding on MeJA- or SA-treated WT and PAP green plants (two comparisons: MeJA-WT vs. MeJA-PAP green, SA-WT vs. SA-PAP green); (II) between nymph feeding on control or MeJA- treated WT and PAP green plants (two comparisons: Control-WT vs. MeJA-WT, Control-PAP green vs. MeJA-PAP green).

## Supporting Information

File S1 **Contains: Protocol S1.** Anthocyanin content of PAP green and WT plants. **Protocol S2.** Quantitative real-time PCR. **Protocol S3.** Chemical characterization of SA and JA levels in WT and PAP green plants. **Protocol S4.** Chemical analyses of nicotine levels in WT and PAP green plants. **Table S1**. List of analyzed phenylpropanoids pathway genes, and their accession numbers, oligonucleodite primers and product sizes. **Table S2.** List of analyzed induced defense pathways genes, and their accession numbers, oligonucleodite primers and product sizes. **Figure S1.** Chemical analyses of nicotine levels in WT and PAP green plants: non-infested (A) or after 5 days of *B. tabaci* infestation (B).
(DOC)
