# Supplementary material for: Activation of the Phenylpropanoid Pathway in Nicotiana tabacum Improves the Performance of the Whitefly Bemisia tabaci via Reduced Jasmonate Signaling
Source: PLoS One. 2013 Oct 25;8(10):e76619. doi: 10.1371/journal.pone.0076619 (PMC3808378; doi:10.1371/journal.pone.0076619)
Supplement: File S1 — Contains: Protocol S1. Anthocyanin content of PAP green and WT plants. Protocol S2. Quantitative real-time PCR. Protocol S3. Chemical characterization of SA and JA levels in WT and PAP green plants. Protocol S4. Chemical analyses of nicotine levels in WT and PAP green plants. Table S1. List of analyzed phenylpropanoids pathway genes, and their accession numbers, oligonucleodite primers and product sizes. Table S2. List of analyzed induced defense pathways genes, and their accession numbers, oligonucleodite primers and product sizes. Figure S1. Chemical analyses of nicotine levels in WT and PAP green plants: non-infested (A) or after 5 days of B. tabaci infestation (B). (DOC) [file pone.0076619.s001.doc]

**Protocol S1.**

Anthocyanin content of PAP green and WT plants

100 mg of fresh weight leaves were extracted in 1mL of methanol with 1% (v/v) HCl. After overnight incubation in the dark at 4 °C with shaking at 150 r.p.m., the extract was centrifuged at 10,500 g for 10 min. The supernatant was transferred to a new tube and served for determination of anthocyanin content using the formula: A_530_ – 0.25A_657_, for subtraction of chlorophyll interference. Ten replicates were conducted for each plant type (WT and PAP green), and the mean values were compared using a Student's *t*-test.

**Protocol S2.**

Quantitative real-time PCR

RNA was extracted from 100 mg of leaf tissue using the TRIzol reagent (Invitrogen), according to the manufacturer’s instructions. Two µg of total RNA were treated with DNaseI (Sigma). Then, first strand cDNA was synthesized by Superscript II (Invitrogen) using 1µg of total RNA and an Oligo-dT primer
(5' TTTTTTTTTTTTTTTTTTTV 3') according to the manufacturer’s instructions. Quantitative real-time PCR (qRT-PCR) reactions were performed using GeneAmp 7300 (Applied Biosystems). The PCR conditions, including the cDNA dilutions and primers concentrations were adjusted for the amplification efficiencies of all genes to be in the log range of 1.9-2.1 and were optimally set to master mix (18 µl) containing: 9 µl Absolute Platinum SYBR green qPCR SuperMix (Invitrogen), 250 nM forward and reverse primers and 2 µl of cDNA diluted 1:10. PCR thermal conditions consisted one cycle of 95 °C for 2 min. followed by 40 cycles of 95 °C for 15 sec. and 60 °C for 1 min. The homogeneity of the PCR products was confirmed by melting curve analyses. The reaction products were collected and ran on 2% agarose gel to verify product size and specificity. Quantification of the transcript level in the WT and PAP green plants was conducted according to the ∆CT method, using actin as the reference gene. The mean of at least three independent biological replicates was calculated. Differences in gene expression (∆CT values) between WT and PAP green plants were tested for significance using the Wilcoxon two-sample test.

**Protocol S3.**

Chemical characterization of SA and JA levels in WT and PAP green plants

Known amounts of plant material (between 90 and 150 mg) were frozen in liquid nitrogen and grounded to a fine powder. Extraction was done by adding 1 mL of 90% methanol and an internal standard (200ng O-Anisic acid dissolved in the extraction solution) to the plant material, followed by sonication for 15–20 min. at room temperature. Samples were then centrifuged at 4000*g* for 1 min. and the methanol was evaporated under a constant nitrogen stream to total dryness. One mL of 5% methanol in PBS was added to the dried samples.

The samples were loaded onto a solid-phase extraction cartridge (Strata-X-A, Phenomenex) containing dimethyl butyl quaternary amine sorbent and washed with
1 mL 25mM ammonium acetate followed by 1 mL methanol. Bounded JA, SA and internal standard were eluted with 1 mL 5% formic acid in methanol. The solvent was evaporated and the residues were silylated with 20 μl BSTFA (N, O-bis-trimethylsillyl-trimfluoroacetamide) and TMS (trimethylchlorosilane) (Supelco).
100 μl hexane were added to the silylated products after which their amount was quantified by gas chromatography (GC).

The chemical identity of JA and SA in plant extracts was verified by GC-MS and also by comparison to standards of each of the tested compounds (Sigma). Compounds quantification was done by peak integration in comparison to the internal standard. Quantitative analyses were conducted by GC (Varian CP 3800) using a VM-5 fused silica column. The column was placed at 40 °C for 1 min. and was then heated by 15 °C min^−1^ to 250 °C (for 5 min.) with nitrogen gas as a carrier, at the flow of 1 mL min^−1^. Further identification of JA, SA and the O-Anisic acid was based on comparison of mass spectra and retention times with those of authentic standards analyzed under similar condition. These standards (after SPE column treatment as described above) were identified in quadropole mass spectrometer (TheromoFinnigan). Helium was used as the carrier gas at flow of 1 mL min^−1^. The standards were tentatively identified using the NIST/EPA/NIH mass spectral library.

**Protocol S4.**

Chemical analyses of nicotine levels in WT and PAP green plants

One-hundred mg of leaf tissue from WT and PAP green plants: non-infested or after 5 days of *B. tabaci* infestation, were harvested and immediately frozen in liquid nitrogen in a 2 mL microcentrifuge. Samples were extracted with 1.5 mL 40% MeOH, containing 0.5% acetic acid, and sonicated for 30 min. in a sonication bath. After centrifugation for 12 min. at 13,000 r.p.m, the supernatant was transferred into a sample vial for high-performance liquid chromatography (HPLC) analyses. HPLC analyses were carried out with Agilent Technologies 1200 series. 5 μl of the samples were injected into Phenomenex Gemini C18 reverse phase column (150 X 2 mm,
5 μm; Phenomenex, Torrance, CA). The solvents were 30 mM NH_4_H_2_PO_4_ and NH_4_CO_3_ (pH 6.4) with 1 mM BuSO_3_Na (solvent A) and MeOH (solvent B). The elution system was as followed: 0-1 min, 10% of B; 1-20 min 95% of B. The column oven was set at 30 ºC at flow rate of 0.5 mL min^−1^. The eluent was monitored at 260, 420, and 100 nm. Nicotine was quantified at 254 nm according to the retention time and UV-vis spectra of a nicotine standard (Sigma). Values represent means of µg Nicotine per g fresh weight (FW) ± standard errors. Nicotine levels in PAP green and WT plants were compared by the Student's *t*-test.

**Table S1**.

| Product size (bp) | Reverse primer sequence (5' to 3') | Forward primer sequence (5' to 3') | Accession number | Gene |
| --- | --- | --- | --- | --- |
| 200 | CACCACCATTCTTGGTCCTC | TTGACAGTGGCTCAAGTTGC | AB008199 | *PALA* |
| 182 | TGACATTCTTCTCACTTTCACCA | TGCTAATGGTGAACTTCATCCA | AB008200 | *PALB* |
| 221 | AGAGGCTTTCCTCATTTCATC | AGAAAAGCCTTGTGGAAGCA | AF311783 | *CHS* |
| 70 | AGGATCACGAACAGTAGCGT | TATTGGCTCTTGGCTTGTCA | EF421430 | *DFR* |
| 120 | ACTCTTCGGGCTTCTTCCAG | CACACATGAACCTTCACGAT | AB236952 | *C4H* |
| 88 | GCAGTATCAGTAGTCCAAGG | GCAAGAACCACTAGAACAGC | U32644 | *TOGT* |
| 145 | TCATCTTCAGTCTGGAGTCT | TTGCTATAGTACGGGAGTGC | AB182580 | *ICS1* |
| 159 | TGCTAAGGGATGCGAGGATG | TGCTCAGTGGTGGCTCAAC | U60495 | *ACTIN* |

**Table S2**.

| Product  size (bp) | Reverse primer sequence (5' to 3') | Forward primer sequence (5' to 3') | Accession number | Gene |
| --- | --- | --- | --- | --- |
| 59 | GCCTTAGCAGCCGTCATGA | GGTCAATACGGCGAAAACC | D90196 | *PR1a* |
| 122 | ATGCAGAGTCCAGCCGTTAT | CCTGCTTATCTTCGCTTGAG | AJ308487 | *AOC* |
| 142 | ACACCTCTGTAATGCCTTCC | TTAACGTCGGATGGTCGCCG | D38123 | *ERF1* |
| 86 | TCCTGACCGAAGTAGTAGCA | CGTTCAGAAGGAGAGACGAT | Z29537 | *PI-II* |

**Figure S1.**
